# Molecular Insight into the Interaction between Epigenetics and Leptin in Metabolic Disorders

**DOI:** 10.3390/nu11081872

**Published:** 2019-08-12

**Authors:** Adam Wróblewski, Justyna Strycharz, Ewa Świderska, Karolina Drewniak, Józef Drzewoski, Janusz Szemraj, Jacek Kasznicki, Agnieszka Śliwińska

**Affiliations:** 1 Department of Medical Biochemistry, Medical University of Lodz, 6/8 Mazowiecka Str., 92-215 Lodz, Poland; 2 Student Scientific Society of the Civilization Diseases, Medical University of Lodz, 251 Pomorska Str., 92-213 Lodz, Poland; 3 Central Teaching Hospital of the Medical University of Lodz, 251 Pomorska Str., 92-213 Lodz, Poland; 4 Department of Internal Diseases, Diabetology and Clinical Pharmacology, Medical University of Lodz, 251 Pomorska Str., 92-213 Lodz, Poland; 5 Department of Nucleic Acid Biochemistry, Medical University of Lodz, 251 Pomorska Str., 92-213 Lodz, Poland

**Keywords:** leptin, DNA methylation, histone modifications, microRNAs, metabolic disorders, obesity, gestational diabetes, insulin sensitivity, fetal programming, leptin resistance

## Abstract

Nowadays, it is well-known that the deregulation of epigenetic machinery is a common biological event leading to the development and progression of metabolic disorders. Moreover, the expression level and actions of leptin, a vast adipocytokine regulating energy metabolism, appear to be strongly associated with epigenetics. Therefore, the aim of this review was to summarize the current knowledge of the epigenetic regulation of leptin as well as the leptin-induced epigenetic modifications in metabolic disorders and associated phenomena. The collected data indicated that the deregulation of leptin expression and secretion that occurs during the course of metabolic diseases is underlain by a variation in the level of promoter methylation, the occurrence of histone modifications, along with miRNA interference. Furthermore, leptin was proven to epigenetically regulate several miRNAs and affect the activity of the histone deacetylases. These epigenetic modifications were observed in obesity, gestational diabetes, metabolic syndrome and concerned various molecular processes like glucose metabolism, insulin sensitivity, liver fibrosis, obesity-related carcinogenesis, adipogenesis or fetal/early postnatal programming. Moreover, the circulating miRNA profiles were associated with the plasma leptin level in metabolic syndrome, and miRNAs were found to be involved in hypothalamic leptin sensitivity. In summary, the evidence suggests that leptin is both a target and a mediator of epigenetic changes that develop in numerous tissues during metabolic disorders.

## 1. Introduction

Metabolic syndrome (MetS) is a cluster of different pathological conditions that are extremely common among modern societies. According to a definition by the World Health Organization (WHO), MetS, which is also called “syndrome X”, is recognized as a combination of insulin resistance (IR), obesity, hypertension, high levels of triglycerides, and low high-density lipoprotein (HDL) cholesterol levels [1]. Studies have revealed that abdominal (visceral) obesity is the third most frequent MetS component among all subjects (from 6.8 to 23.6%) [1,2]. Moreover, hypertrophy of visceral adipose tissue (VAT), but not subcutaneous adipose tissue (SAT), is an independent risk factor for the most common obesity-associated disorder, type 2 diabetes (T2DM). T2DM, together with IR, are accompanied by low-grade chronic inflammation, defective insulin signaling and function of the β-cells as well as a disturbance in the production and action of adipocytokines such as adiponectin, resistin, interleukin 6 (IL-6), leptin, and more [3,4].

Adipocytokines are bioactive peptides produced by adipose depots that play an essential role in the regulation of insulin signaling, glucose transport, lipid metabolism, and inflammation [4]. Leptin is a hormone exhibiting an anorexic effect, whose discovery confirmed that adipose tissue (AT) is an endocrine organ involved in energy metabolism [5]. Obesity, T2DM, and MetS have been found to be associated with deregulated serum leptin levels, while leptin resistance has been recognized as an important factor in obesity [6,7,8,9]. Furthermore, current data suggest that the environment triggers epigenetic modifications that are strongly involved in the formation and progression of obesity-related diseases [10]. Therefore, in this review, we focused on recent achievements in the field of the epigenetic regulation of leptin as well as leptin-induced epigenetic phenomena in metabolic disorders such as the adipogenesis, fetal, and early postnatal programming of metabolism.

## 2. Leptin

### 2.1. Structure

Leptin is one of the most relevant cytokines produced by adipocytes and is a 167-amino acid polypeptide encoded by LEP(Ob) gene [11]. The human LEP gene is located on the q arm of chromosome 7, band 32, sub-band 1 [12]. The structure of this protein hormone is similar to pro-inflammatory helical cytokines (it comprises a four-helical core) and contains one disulfide bond that is essential for binding to leptin receptors [13,14]. While circulating in the bloodstream, leptin binds to serum macromolecules, suppressing its availability for receptors, whereas a minor amount of leptin remains unbound [13].

### 2.2. Secretion

Leptin is secreted into the bloodstream by adipocytes proportionally to the size of AT or adipocyte (SAT is a major producer of this hormone), and postprandially. Accordingly, insulin is a key factor that triggers leptin secretion and transactivates the LEP gene, while glucagon and catecholamines inhibit both of these processes. Leptin’s mRNA is also increased by glucose, glucocorticoids, tumor necrosis factor alpha (TNF-α), and the CCAAT-enhancer-binding protein alpha (C/EBPα) transcription factor. However, inhibited transcription of LEP is elicited by, among others, free fatty acids (FFA), growth hormone, and the receptor agonists peroxisome proliferator-activated receptor-γ (PPARγ) and liver X receptor (LXR) [15,16]. Except for AT, leptin is also expressed in the placenta, hypothalamus, bone marrow, and stomach. Additionally, it is secreted in both a paracrine and endocrine manner [13,15].

### 2.3. Functions

Leptin acts on a variety of target tissues since the presence of the leptin receptor (LEPR) is common. There are six alternatively spliced isoforms of LEPR. Commonly, LEPR consists of extracellular (800 amino acids), transmembrane (34 amino acids), and intracellular domains, whereas the last is specific for each isoform [13]. Depending on the length of the intracellular domain, LEPR isoforms divide into short, long, and LEPRe isoforms lacking the intracellular domain, where the latter is considered as a secretory receptor. Therefore, LEPRe may bind circulating leptin, thus serving as a carrier for leptin and regulating its blood concentration. Short isoforms contain only a box 1 motif in their intracellular domains, however, long LEPRb receptors contain three additional tyrosine residues phosphorylated for further signaling. Indeed, LEPRb is considered as an isoform mediating the main biological effects of leptin [11].

The basic role of leptin is to transmit the satiety state signal to the hypothalamus [15]. LEPRa and LEPRc localized in the choroid plexus take part in the uptake of leptin from the cerebrospinal fluid and in the transport across the blood–brain barrier (BBB) [13]. Leptin stimulates the expression of pro-opiomelanocortin (POMC) in POMC-expressing hypothalamic neurons, which then increases energy expenditure by α-melanocyte-stimulating hormone (α-MSH) activity. Additionally, leptin Agouti-related protein (AgRP)-expressing neurons cease the expression of orexygenic neuropeptide Y (NPY) and AgRP, thus decreasing hunger and restoring α-MSH activity [17]. Hypothalamic leptin activity also induces the neuroendocrine stimulation of the thyroid, gonads, and suppression of adrenal activity, hence modulating energy expenditure [16]. Moreover, in peripheral tissues, leptin improves glucose transport, glycolysis, and insulin sensitivity. It also affects the expression of enzymes crucial for lipid metabolism (e.g., fatty acid synthase). As a result, the biosynthesis of fatty acids in the liver, skeletal muscles, and AT is restrained and β-oxidation in the skeletal muscles and heart muscle is enhanced [15]. Therefore, leptin regulates the intake and expenditure of energy depending on the available resources. Moreover, a wider range of effects is exerted in tissues expressing LEPRb, which is able to activate various signaling pathways including the JAK/STAT, SHP2/ERK, IRS/PI3K, and MAPK cascade [13,17].

Other effects of LEPRb activation concern the inflammatory response and increased production of Th1 cytokines (e.g., TNF-α, IL-6) along with suppressed expression of Th2 cytokines. On the other hand, leptin’s expression is also elevated by inflammation [14]. In addition, through LEPRb activity, leptin also regulates bone, cardiovascular, reproductive function, and angiogenesis [13].

In particular, the relevance of insulin in the regulation of leptin levels and involvement in insulin downstream signaling indicates the key role of leptin in obesity-induced IR, which is under the thorough consideration of scientists.

Commonly, a higher level of leptin follows obesity, however, leptin appears to be ineffective against excessive AT accumulation. Growing evidence suggests the existence of a leptin resistance phenomenon that could occur as a distortion of the transport or signaling of leptin [17]. Impaired transport across the BBB, endoplasmic reticulum (ER) stress, obesity-induced chronic inflammation, and hyperleptinemia-induced suppressor of cytokine signaling 3 (SOCS3) transactivation are regarded as factors of leptin resistance [16]. Chronic stimulation of LEPRb results in the overexpression of SOCS3, a protein capable of attenuating leptin signaling, and is usually considered as leptin negative feedback mechanism [13]. Another interesting molecule is a non-receptor protein tyrosine phosphatase 1B (PTP1B), which performs dephosphorylation of Janus kinase 2 (JAK2), thus repressing leptin signaling. Moreover, studies involving a HFD (high-fat diet) treatment show that PTP1B vastly contributes to insulin and leptin resistance [18]. Since leptin is a pro-inflammatory adipokine and hyperleptinemia in leptin resistance may induce chronic inflammation, future research should fully elucidate the role of leptin resistance in the pathogenesis of metabolic disorders. Considering the dysregulation of the BBB leptin transport and imbalance between free and bound fractions of circulating leptin is also worth consideration. Importantly, the leptin in cerebrospinal fluid is only modestly elevated in obesity independent of hyperleptinemia [13,17]. Moreover, ER stress is well-known to be involved in the development of metabolic disorders. Hypothalamic ER stress also seems to be followed by leptin resistance, thus further research should give a better understanding of this relationship as well as for other possible patomechanisms of leptin resistance [17].

## 3. Mechanisms of Epigenetic Modifications

Epigenetic modifications are ones that are (i) triggered by environmental exposure, (ii) not associated with changes in DNA sequence, and (iii) result in the acquisition of heritable traits [18]. Epigenetics involves changes of DNA accessibility (de/methylation), the structure and dynamics (posttranslational histones modifications) of chromatin, miRNA interference, and modifications of RNAs such as methylation [19]. The mentioned mechanisms of DNA methylation and demethylation, are processes governed by DNA methyltransferases (DNMTs) and DNA demethylases. DNA methylation is performed via the covalent addition of the methyl group to cytosines within CpG dinucleotide motifs. Methylated DNA is typically detected for regions with lower transcriptional activity and repressive a chromatin state. It is accompanied by the binding of specialized proteins (methyl-CpG binding proteins), impaired accessibility of transcription factors to gene expression regulatory elements, and inhibitory posttranslational modifications of N-terminal tails of histones [20]. Core histone tails can be modified via acetylation, methylation, ubiquitination, phosphorylation, and sumoylation at arginines, lysines, and histidines, which affect amino acid non-covalent interactions and chromatin folding [21]. However, the acetylation and methylation of lysines and arginines still appear to be of top interest for gene expression studies. The latter processes are mediated by histone methyltransferases (HMTs) and demethylases (HDMs) as well as histone acetyltransferases (HATs) and deacetyltransferases (HDACs) [10]. Acetylation of lysine-9/14 sites within H3 histone is a hallmark of transcriptional activation, while the outcome of histone methylation depends on the degree of methylation (mono-, di- or tri-methylation) and location of the methyl lysine residues [21]. For instance, di/trimethylation of lysine-4 and lysine-79 of the H3 histone (H3K4me2/3 and H3K79me2/3) has been demonstrated to activate transcription, yet, H3K9me3, H3K27me3, and H4K20me3 have been reported to repress gene expression [21,22,23]. Another mechanism, microRNA (miRNA) interference, involves the action of short, approximately 22 nt-long, non-coding RNAs, whose biogenesis is a multistep process occurring in both the nucleus and cytoplasm [24]. Each miRNA is capable of regulating the expression of hundreds of mRNAs, thus have an enormous impact on both the transcriptome and proteome [24]. The most well-studied function of miRNAs is the posttranscriptional negative regulation of target mRNAs, which are recognized via a Watson–Crick pairing in the seed region, providing RISC is formed (RNA-induced silencing complex) [24]. miRNA-RISC acts in cytoplasm either via hindering translation or degrading target mRNAs, however, there is also increasing evidence of its activity in the nucleus [24]. Moreover, miRNAs are suggested to act in the nucleus independent of RISC, while affecting alternative splicing or performing transcriptional activation or repression of gene expression [24]. Last but not least, RNA methylation has recently gained interest as a novel form of epigenetic regulation [25]. Until now, there are 72 reversible methyl-specific modifications of RNAs, however, only a few of them have been found on mRNAs [24]. N6-methyladenosine (m6A), the most common mammalian modification of mRNA, occurs around stop codons, within internal long exons, or in untranslated regions (3′UTRs) [26]. This methylation mark is “written” by the multicomponent methylation complex, displaced (“erased”) by demethylases, and read by specialized proteins mediating changes in RNA maturation, translation, degradation, or even splicing [27]. An accumulating amount of data suggests the strong interplay between all known components of epigenetic machinery, forming a so-called miRNA-epigenetic feedback loop to affect gene expression and take part in the development of various diseases [28]. Except for RNA methylation, all other types of epigenetic mechanisms have been demonstrated as deregulated in multiple tissues by inflammation, obesity, IR, T2DM, metabolic syndrome, gestational diabetes, or in the context of adipogenesis and fetal metabolic programming [29,30,31,32,33,34,35].

## 4. Epigenetic Regulation of Leptin’s Expression in Metabolic Disorders

### 4.1. DNA Methylation

To our best knowledge, variations in LEP methylation in patients with metabolic disorders have been studied in saliva, blood, and AT. In saliva, sex-stratified analysis with adjustment for age showed that percentage of body fat, BMI, and waist circumference were negatively associated with four CpG sites-measured LEP methylation in obese boys, but not girls, in their mid-childhood [36]. Analysis involving adjustment for age and parental BMI also showed an association between lower LEP methylation levels and obesity in these boys [36]. Consistently, reduced frequency of methylation of the CpG site (−51 and −31 nt) in the LEP promoter was found in the peripheral blood of obese and morbidly obese adolescents (10–16 years, both sexes) with IR [37]. Similar results were obtained by Sayed et al., who postulated that obese children exhibit whole blood-specific LEP hypomethylation when compared to both age-sex matched group with normal weight as well as to an obese follow up group that experienced folic acid intake [38]. Interestingly, this study also showed that supplementation of folic acid potently ameliorates obesity-associated reduction in LEP methylation [39].

Considering obese adults, Houde et al. presented that CpG methylation status was positively correlated between blood and SAT in of six out of 21 analyzed CpG sites within the LEP promoter as well as the methylation of two analyzed CpG sites that showed the same correlation between VAT and blood [39]. Specifically, the methylation of CpG11 was indicated as highly correlated between VAT and SAT as well as SAT and blood, while multiple testing correction also showed the trend for the association between VAT and blood [39]. Another CpG site, CpG17, correlated between blood and SAT [39]. The same group reported some positive correlation between LDL cholesterol, a factor known to indirectly modulate DNA methylation, and the methylation levels of LEP-CpG11 and -CpG17 in paired samples of blood and SAT, but not VAT [40]. Moreover, LEP methylation in white blood cells was negatively associated with BMI [40]. Having measured the expression and methylation of LEP in AT and liver, Merchi et al. stated that promoter methylation regulated tissue-specific expression of leptin rather than serve as a mechanism controlling leptin’s expression in the white AT of severely obese patients experiencing bariatric surgery [41]. However, baseline LEP methylation, but not expression of leptin, was indicated to be a good predictor of response to dietary intervention in women, being much lower in the responder group [42]. In contrast, LEP methylation of four studied CpG sites in the VAT samples did not reflect differences between patients with and without MetS, yet showed a positive correlation with blood pressure [43].

In studies conducted on animal models, LEP promoter methylation on one out of 13 tested CpG sites (−443 position) in retroperitoneal adipocytes was increased in male Wistar rats fed on a high-fat diet in comparison to animals on a standard diet, which was concurrent with lower levels of circulating leptin [44]. A study performed on a HFD-induced mice model showed that the LEP promoter was hypermethylated in gonadal AT, but not in SAT [45]. Another study on HFD-fed animals indicated that leptin’s expression, the methylation of leptin’s promoter, and binding of MBD (methylated DNA binding domain) proteins, RNA polymerase II and DNMTs along with the expression of DNMTs in AT, were subjected to time-specific changes during the development of obesity [46]. Specifically, the methylation level of the leptin promoter was downregulated after eight weeks of a HFD, but was upregulated after 12 and 18 weeks of such exposure [46]. Such pronounced and various changes of methylation-associated parameters clearly indicate that the development of obesity is connected with the sophisticated orchestration of biological events associated with leptin regulation [46]. On the other hand, Okada et al. did not report changes in LEP promoter methylation in the WAT of C57BL/6J mice treated with a HFD, although the upregulation of leptin was observed [47].

To summarize, the collected data suggest that the leptin promoter is hypomethylated in metabolic disorders as well as also being correlated with some important biochemical and anthropometric parameters or serve as a potent marker of response to dietary intervention.

### 4.2. Postranscriptional Histone Modifications

Various histone modifications of the leptin promoter have been explored in the AT of DIO (diet-induced obese) mice model with and without treatment with n-3 PUFA (polyunsaturated fatty acids), which are known to reduce body fat [48]. Specifically, the acetylation levels of histones H3 and H4 and methylation of H3K4 were significantly reduced and no changes in the methylation of H3K9/27 were observed in DIO mice when compared to the control animals. Paradoxically, these changes were accompanied by (i) an elevation of circulating leptin and mRNA leptin expression in AT, (ii) higher methylation of nine out of 16 tested promoter CpGs, (iii) increased binding of HDAC1/2/6, DNMT1, DNMT3a, DNMT3b, and MBD2, and (iv) decreased binding of RNA Pol II to the leptin promoter in AT [48]. Such a lack of compatibility among the expression level, binding of epigenetic enzymes, and histone modifications only underlies the complexity of the mechanisms regulating the expression of leptin in obesity. Moreover, n-3 PUFA decreased the level of circulating leptin and its mRNA expression in AT. However, n-3 PUFA (i) had no impact on CpGs methylation, (ii) triggered significant reduction of the binding of only MBD2 and DNMT1, (iii) increased the acetylation of H3 and methylation of H3K4/9, (iv) suppressed the binding of HDAC1/2/6, and (v) did not affect the binding of the CREB-binding protein to the leptin promoter in AT [48]. It is suggested that n-3 PUFA acts like an antagonist against modification of the leptin promoter mediated by a DIO diet. Moreover, it has also been hypothesized that the strong methylation of H3K9, known to firmly promote heterochromatin formation along with other repressive molecular events, could be the most dominant way of mediating leptin downregulation by n-3 PUFA, which could override all other studied mechanisms. To our best knowledge, this comprehensive and impressive study is the only one showing histone modifications within the leptin promoter during metabolic imbalance induced in adulthood so far.

### 4.3. miRNAs

**Figure 1:**
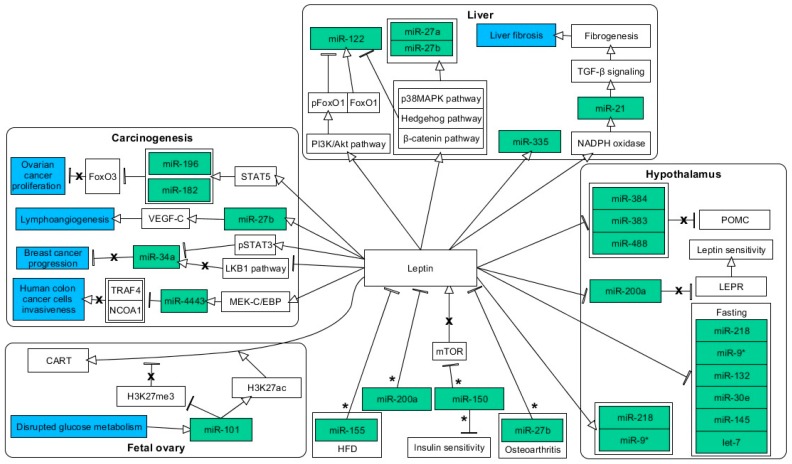
Proposed model of miRNA participation in downstream signaling and regulation of leptin expression. miRNAs and negative outcomes are highlighted in green and blue, respectively. Associations are indicated by arrows (stimulation) and T-bars (inhibition). Actions related to boxes refer to all items inside the box. Arrows and T-bars which are crossed out mark the abrogation of the respective type of regulation. Associations were based on the leptin administration or the ob/ob model excluding those marked by asterisks (exclusively, the effect of miRNAs on leptin expression was measured, without prior manipulation of the leptin level). The figure was made using the PathVisio 3.3.0 free open-source software [53].

The data regarding miRNA-dependent direct regulation of leptin in cell, animal, and human models of metabolic disorders appear to be highly limited, which may be due to the fact that animal models of obesity and diabetes frequently involve gene knockouts of leptin or leptin receptors. However, there are studies concerning the indirect impact of some miRNAs on the production or expression of leptin. First, miR-155 has been indicated to suppress the HFD-induced increase of plasma leptin concentration in apoE−/− mice [49]. Moreover, mice with knockout of miR-150 showed reduced body weight and elevation of leptin concentration, along with improved glucose tolerance and insulin sensitivity [50]. As suggested by the authors, this effect could have been mediated by the lack of inhibitory impact of miR-150 on mammalian target of rapamycin (mTOR) expression, which is known to participate in leptin production [50]. Considering examples of direct interaction between miRNAs and leptin, the latter has been proved to be regulated by miR-27 in osteoarthritic chondrocytes and miR-200a/b in Yellow Catfish [51,52]. These studies indicate that leptin is a possible target for miRNA interference during metabolic imbalance (Figure 1).

## 5. Leptin-Induced Epigenetic Modifications in Metabolic Disorders

### 5.1. DNA Methylation

The impact of leptin on global DNA methylation in inguinal adipocytes has been suggested in studies performed on ob/ob and DIO C57BL/6J mice [54]. Future studies regarding leptin-mediated changes of DNA methylation are warranted, as ob/ob mice have been reported to exhibit expression changes of DNA methyltransferases (DNMT1/3a/3b) in AT [55,56].

### 5.2. Postranscriptional Histone Modifications

Leptin, or leptin sensitivity, appears to increase the expression of HDAC5 in the hypothalamus of ob/ob mice, based on the results obtained for animals treated with leptin during ad libitum feeding in comparison to saline-treated ones [57]. Importantly, knockout of HDAC5 caused mice to be more prone to diet-induced obesity [57]. Another study showed that ob/ob and db/db mice exhibited a reduction in sirtuin 1 (SIRT1) expression, a histone deacetylase, in comparison to C57BL/6J mice [58]. Furthermore, a great body of research is now focused on obesity-related carcinogenesis, which occurs due to, among others, hyperleptinemia [59]. For instance, the expression of SIRT1 was upregulated via leptin-induced, nuclear factor, erythroid 2 Like 2 (Nrf2)-dependent signaling in colon cancer [58]. Leptin was also found capable of modulating HDACs, and thus inducing progression and chemoresistance of pancreatic carcinoma [60]. All of these findings should pave the way for the novel area of research in the context of metabolic disorders.

### 5.3. miRNAs

The impact of leptin on miRNAs has been studied in several tissues including the hypothalamus. First, pharmacological blockage of leptin during early life (part of the weaning period) triggered differential expression of 38 miRNAs at the end of this period [61]. Among these miRNAs, the expression of rno-miR-200a, rno-miR-409-5p, and rno-miR-125a-3p in the hypothalamus was changed by a month-long HFD treatment, which started about 100 days after the end of weaning, and was concurrent with excessive weight gain in comparison to the control animals. These results imply that leptin signaling during early life is critical for the expression of miRNAs in the hypothalamus and vastly affects metabolism in adult life. Moreover, the administration of leptin in adult male rats during fasting or ad libitum feeding caused differential expression of hypothalamic let-7, miR-132, mir-145, miR-9*, mir-218, and miR-30e [62]. In addition, miR-132 was shown to stimulate hippocampal synaptogenesis through leptin-induced cAMP response element-binding protein (CREB) phosphorylation [63]. Other data showed that ob/ob and db/db mice exhibited hypothalamic upregulation of mir-383, mir-384-3p, and mir-488, the proven negative regulators of POMC expression [64]. Further studies showed that leptin injected intraperitoneally caused their downregulation in ob/ob mice, and that intracerebroventricular chronic injection reduced the level of miR-488 in C57BL/6 mice. Thus, leptin appears to control miRNAs involved in the regulation of POMC in the hypothalamus during metabolic imbalance [64]. Another miRNA, mir-200a was subjected to reduction upon treatment with leptin in ob/ob mice, while its silencing in the hypothalamus increased the expression of leptin receptors, decreased body weight, and restored liver insulin sensitivity [65]. Interestingly, results obtained based on animals with partial deletion of the miRNA processing enzyme DICER indicated that miRNAs may participate in the regulation of hypothalamic leptin sensitivity [66].

Obesity, apart from diabetes and atherosclerosis-related cardiovascular disease, makes an excellent background for liver disorders. One of them, NAFLD (nonalcoholic fatty liver disease), is a broad-spectrum disorder including steatosis and NASH (nonalcoholic steatohepatitis), which may proceed to liver fibrosis, cirrhosis, and finally, hepatocellular carcinoma [67]. High leptin levels were suggested to be an additional risk factor for liver disorders [68,69]. In NASH, leptin was reported to upregulate NADPH oxidase, thus leading to the induction of miR-21 and consequent transforming growth factor beta (TGF-β) signaling-mediated augmentation of fibrogenesis [69]. Leptin was also indicated to utilize the NADPH oxidase 2-miR-21 axis to promote NAFLD-related systemic inflammation such as renal inflammation [70]. Considering liver fibrosis, incubation with leptin caused indirect upregulation of mir-27a/b in HSCs (hepatic stellate cells), whose activation is a critical step in fibrosis, based on results obtained in vitro and in vivo (C57BL/6J ob/ob mice), via at least three different molecular pathways (β-catenin, Hedgehog, and p38MAPK) [68]. Moreover, leptin was proven to downregulate miR-122 in HSCs also via the Hedgehog pathway, while miR-122 abolished leptin-mediated liver fibrosis in ob/ob mice [71]. Further studies revealed that leptin indirectly decreased promoter activity of miR-122 via inducing Forkhead box protein O1 (FoxO1) phosphorylation at serine 256 via the PI3K/Akt pathway [72]. When FoxO1 was unphosphorylated, it increased levels of miR-122 and suppressed leptin-dependent HSC activation as well as liver fibrosis in ob/ob mice [72]. There have also been some studies suggesting the impact of leptin on miRNAs via the ob/ob model. For instance, studies on these animals indicated liver and AT-localized upregulation of miR-335, while the stimulatory impact of leptin on miR-335 was then confirmed in human mature adipocytes [73,74].

As above-mentioned, obesity together with hyperleptinemia is a risk factor for the occurrence of various types of cancer. For instance, stimulation of leptin elicited signal transducers and activators of transcription-5 (STAT5)-mediated increase of miR-182 and miR-196, which were proven to target forkhead box O3 (FOXO3), thus leading to augmentation of ovarian cancer proliferation [75]. Furthermore, leptin enhanced lymphoangiogenesis by triggering the production of vascular endothelial growth factor C (VEGF-C) in human chondrosarcoma cells [76]. This biological effect was a result of the leptin-induced expression increase of miR-27b [76]. In breast cancer, leptin showed an oncogenic effect via downregulating miR-34a either by repressing the liver kinase B1 (LKB1) pathway or due to the phosphorylation of STAT3 [77,78]. In contrast, miR-4443 expression was elevated upon leptin administration via mitogen-activated protein kinase kinase (MEK)-C/EBP to reduce the expression of nuclear receptor coactivator 1 (NCOA1) and TNF receptor-associated factor 4 (TRAF4) [79]. Activation of this signaling axis resulted in the amelioration of the invasiveness of human colon cancer cells [79]. The general pro-oncogenic influence of leptin and other adipokines was also indicated in a recent review by Jasinski-Bergner et al. [80]. Altogether, an accumulating amount of data suggest that leptin is a potent indirect regulator of miRNA expression in tissues critically affected by obesity and during carcinogenesis (Figure 1).

In addition to the above-mentioned miRNAs, some have been shown to be linked to leptin based solely on correlation analysis. Klöting et al. found that blood leptin was correlated with the expression of miR-17, miR-132, miR-99a, miR-134, miR-181a, miR-145, and miR-197 in both human omental and subcutaneous AT [81]. In morbidly obese adolescents, circulating miRNAs such as miR-222, miR-143, miR-142-3p, miR-140-5p, and miR-130 were elevated, whereas miR-15a, miR-146a, miR-423-5p, miR-520c-3p, and miR-532-5p were declined in comparison to the controls. However, all of them were positively associated with circulating leptin levels [82].

## 6. Epigenetics of Leptin in Adipogenesis

Since leptin is an adipocyte effector protein, its expression is clearly equated as a marker of preadipocyte differentiation [83]. To become a mature adipocyte, preadipocytes go through commitment and maturation phases. The former step is a genetic preparation of fibroblast-like preadipocyte to adipocyte duties, and in the latter step, adipocytes start to store lipid droplets, express its characteristic cytokines (adipokines), and secrete them [84].

### 6.1. DNA Methylation

Since 2002, scientists have indicated a decreased methylation of CpG islands placed in the LEP promoter region during the differentiation of human preadipocytes to leptin-expressing adipocytes. LiSa-2 cells treated with azacitidine (5-aza-dC), which acts as a demethylating agent, showed an analogous decline in LEP promoter methylation. Methylation of specific CpG in the C/EBPα binding site markedly restrained leptin promoter activity and binding of transcription factors in LiSa-2 cells as well as the methylation of the Sp1 binding site, despite the a decreased effect [85]. Correspondingly, another study demonstrated the demethylation of all CpGs in the fragment of the mouse LEP promoter ranging from positions −54 to −159 during the differentiation of 3T3-L1 preadipocytes excluding the CpG dinucleotide in the C/EBPα binding site. Furthermore, the luciferase reporter assay indicated that leptin promoter activity was not affected by the methylation of this CpG, whereas the methylation of the CpGs within the Sp1 binding site reduced promoter activity [86]. Additionally, Noer et al. observed at most a 32% level of CpG-specific methylation within the LEP promoter of cultured undifferentiated adipose stem cells (ASCs). Particular clones of ASCs showed heterogenous methylation of leptin promoter CpGs, which was unrelated to the expression of leptin. Nonetheless, the methylation of CpGs in the C/EBPα binding site substantially decreased leptin expression. Although global methylation remained unchanged after the differentiation of ASCs, researchers observed a significant decrease in the methylation of a few particular CpGs [87]. Differentiated ASCs also showed unmethylated sites within SP-1 and C/EBPα binding regions on the LEP promoter, which was consistent with the data of Melzner et al. [81,83]. Further research on senescence-induced methylation in ASCs support the role of C/EBPα binding site methylation in the regulation of leptin’s expression [88].

In summary, many research reports have highlighted the pivotal role of C/EBPα binding in leptin’s expression. In adipogenesis, the differentiation process prepares the cell to express its effector proteins. Therefore, the opening of the transcription factors’ binding sites by demethylation also seems to be an element of such preparation and has been suggested to reflect the commitment of the cell to a specific lineage.

### 6.2. Postranscriptional Histone Modifications

Chromatin decondensation is a fundamental condition for gene expression. It has been shown that the acetylation and methylation state of histones influences the preadipocyte differentiation along with the expression of leptin [89].

The relationship of leptin and histone acetylation was partially evidenced in 2004, when acute treatment with valproic acid (VPA) downregulated the leptin mRNA level in 3T3-L1 adipocytes, but showed unchanged expression after trichostatin A (TSA) treatment, opposed modulation through HDAC [90]. In another study, treatment with HDAC inhibitors (HDACi) decreased preadipocyte differentiation and leptin secretion in human SAT [83]. The former study revealed an intricate impact of HDACi on leptin mRNA in differentiated adipocytes, whereas the latter study suggests that HDACi-inhibited differentiation resulted in lower leptin expression. Consistently, Yoo et al. indicated that during the differentiation of 3T3-L1 cells, expression of HDAC1, HDAC2, HDAC5, and HDAC6 declined, along with increased overall histone acetylation. Correspondingly, HDAC activity was lower in adipocytes than in preadipocytes and the knockdown of HDAC1 stimulated adipogenesis [91]. Therefore, the acetylation of histones seems to be necessary for adipogenic differentiation, which is consistent with HDACi-mediated disruption of preadipocyte differentiation. Yoo et al. also suggested that the early stage of preadipocyte differentiation is a pivotal moment for chromatin remodeling [91]. On the other hand, Musri et al. found that H3 acetylation and H3K4 trimethylation of the LEP promoter occurred during the differentiation of 3T3-L1 cells excluding leptin non-expressing 3T3-L1 fibroblasts. However, H3K4 dimethylation has already been detected in 3T3-L1 fibroblasts, marking a commitment to s adipogenic lineage [89].

Consequently, a relationship between leptin expression and histone acetylation remains entangled in adipogenic differentiation and early chromatin remodeling.

### 6.3. miRNAs

While the regulation of leptin expression by microRNAs (miRNAs) is mostly unknown, many recent reports have indicated miRNAs affected by leptin. In a few recent studies, leptin induced the expression of miR-199a, miR-335, miR-21, and miR-378 in human mature adipocytes [76,92,93,94]. Moreover, the expression level of miR-1908 was decreased in human mature visceral adipocytes up to 24 h after incubation with leptin [95], and miR-221 was subjected to leptin-induced downregulation in primary human mature adipocytes [96]. Furthermore, researchers observed increased expression of miR-199a, miR-335, and miR-378 during the differentiation of human mature adipocytes, whereas the elevation of miR-21 was transient [76,92,93,97]. Results suggest that these miRNAs are involved in adipocyte metabolism and preadipocyte differentiation. Bioinformatics analysis indicated that miR-335 takes part in adipogenesis, lipogenesis, and fatty acid metabolism, whereas miR-199a was indicated as a mediator of IR and inflammation [74,92]. Zhang et al. implied that miR-21 contributes to obesity-related IR, as pro-inflammatory adipokines related to this metabolic disorder (e.g., resistin and TNF-α) have a similar effect on miR-21 expression [94]. By this set of miRNAs, leptin may trigger a whole machinery to regulate adipocyte metabolism, and their dysregulation could potentially lead to IR, chronic systemic inflammation, and metabolic syndrome. Further studies should clarify whether hyperleptinemia could trigger detrimental metabolic changes through excessive expression of these miRNAs.

Leptin, along with other adipokines, had a negative effect on the expression of miR-26b in human mature adipocytes, according to a study from 2013 [98]. miR-26b was previously shown to be induced during the differentiation of adipocytes in several reports concerning human and murine cell lines [98,99,100]. Furthermore, knockdown experiments suggested a regulatory role of this miRNA in predipocyte differentiation, which was supported by a lower expression of core adipogenesis regulatory genes (PPARγ and C/EBPα) [99]. miR-26b is especially worth attention since its predicted target, phosphatase and tensin homolog (PTEN), is involved in the regulation of cell cycle and insulin sensitivity. Direct action of miR-26b on PTEN was repeatedly confirmed by reporter luciferase assay [99,101]. Therefore, abundant secretion of leptin in obesity could affect miR-26b to limit the preadipocyte differentiation process through PTEN, and PPARγ and C/EBPα, however, the detailed mechanism remains to be fully elucidated and clarified. Furthermore, meta-analysis performed by Liang et al. proposed a group of miRNAs (significantly dysregulated in T2DM) including miR-26b as potential circulating biomarkers of T2DM [102]. As for miR-143, Chinese researchers have reported on leptin’s inhibitory effect on this miRNA in human mature adipocytes, suggesting a negative feedback action of this adipokine [103]. miR-143 was evidenced to be involved in adipogenesis due to the induction of its expression during the differentiation of both human and murine preadipocytes [100,104]. Additionally, in bovine intramuscular adipocytes, miR-143 shows a similar expression pattern, however, a lack of this miRNA inhibits preadipocyte differentiation and promotes preadipocytes proliferation. Knockdown of miR-143 also resulted in a decrease of C/EBPα expression and fatty acid-binding protein 4 (FABP4) expression [105]. Aside from the regulation of adipogenesis rate, study on murine liver cells revealed that overexpression of miR-143 disrupted insulin-dependent AKT activation, implying the involvement of this miRNA in obesity-induced IR [106]. Thus, miR-143 and miR-26b could belong to epigenetic switches (more complicated mechanism) by the reduction of which leptin controls the adipogenesis and energy storage. Moreover, miR-143 is definitely involved in obesity-induced IR, since overexpression of this miRNA can cause IR. However, contribution of miR-143 to the formation of this metabolic disorder is still unclear, seeing that obesity comes with higher leptin secretion, and the complexity of the leptin resistance phenomenon. Therefore, further research in this area is necessary to elucidate the role of miR-143 in the genesis of IR with reference to the relationship with leptin resistance mechanisms.

**Figure 2:**
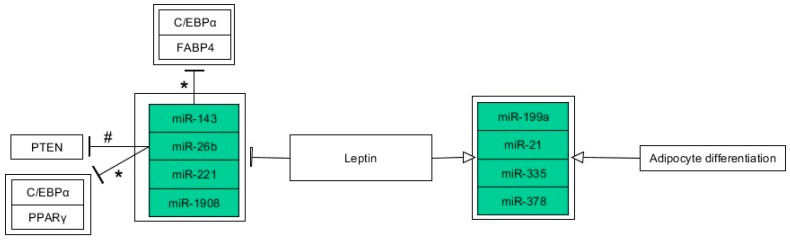
Leptin-mediated miRNA expression changes in adipocytes and during adipogenesis. miRNAs are highlighted in green. Associations are indicated by arrows (stimulation) and T-bars (inhibition). Actions related to boxes refer to all items inside the box. Associations were based on the results of the studies involving leptin administration excluding those marked by asterisks (miRNA knockdown experiments) or hash (targeting proved by reporter luciferase assay). The figure was made using the PathVisio 3.3.0 free open-source software [53].

To sum up, miRNAs that were found to be related to leptin are seemingly involved in the regulation of adipocyte metabolism and differentiation (Figure 2). In the research on AT and leptin, adipogenesis is a narrow area among other available research models. Recently, fetal programming of metabolism is a novel and further concern for this area of research.

## 7. Epigenetics of Leptin in Fetal Programming

Fetal programming is a phenomenon affecting in utero processes, which alters metabolism in later life, and thereby contributes to the development of plenty of disorders [107]. Metabolic programming involves epigenetic changes not only of the fetus, but also within the placenta [108]. While it is well-known that any insult including stress, famine, gestational diabetes, excess pregnancy weight gain, or even high-fructose diet may have a sustained impact on the intrauterine milieu and the development of the fetus, it appears that even paternal obesity plays a role [109,110,111].

### 7.1. DNA Methylation

In 2013, Lesseur et al. suggested that factors such as newborn gender, birth weight, and pre-pregnancy obesity affected methylation of the LEP promoter, depending on the tissue. In the placenta, methylation of the LEP promoter differed among newborn gender (higher in male infants), however, it was not found to be correlated with birth weight. Despite this, newborns small for gestational age (SGA) had higher cord blood LEP methylation [112]. In contrast, DNA methylation of the LEP gene in SGA newborns and normal postnatal growth newborns did not differ in blood collected at the age of 19 [113]. More recently, a study on ASCs that were isolated from the SAT of adult individuals revealed a correlation between birth weight and genome-wide DNA methylation in a group born with low birth weight (LBW). However, no such correlation was found afterward in differentiated mature adipocytes [114]. Thus, fetal programming might affect DNA methylation, especially in progenitor cells, which is consistent with the previously suggested attenuation of the programmed methylation changes over the years and cell differentiation [112,113]. On the other hand, genome-wide methylation pattern may differ from specific promoter methylation, therefore, further studies should concentrate on the methylation of specific promoters to verify this association in pivotal adipogenic genes such as LEP.

More lately, Wang et al. reported a case-control study in which newborns with non-gestational diabetes (non-GDM) macrosomia had lower average LEP methylation in cord blood, whereas methylation of 11 CpGs correlated with macrosomia. Besides a low methylation of the LEP, for example, high cord blood leptin levels and high pre-pregnancy BMI, and male gender were contributing factors of macrosomia [115]. Thus, we can hypothesize that in early life, cord blood LEP promoter methylation is programmed inversely to birth weight.

As for the dynamics of leptin production and secretion, Hjort et al. indicated different results of 36-h fasting episodes depending on the birth weight of the adult participants. Methylation of LEP promoter in the SAT was higher in LBW when compared to normal birth weight (NBW) individuals before fasting, whereas the fasting episode increased methylation only in NBW. Methylation was also correlated with total body fat percentage. Leptin serum levels were higher in LBW adult men before fasting and dropped in both groups respectively [116]. Leptin secretion and methylation of its gene promoter responses in both groups were unaffected by birth weight, however, apart from the fasting episode, the data suggest the putative contribution of birth weight to metabolic dysregulation. The authors indicated that basic LEP methylation in SAT was higher in LBW adults, which argues with previously mentioned reports of isolated ASCs. These discrepancies may be explained by the disproportion between ASCs and whole SAT tissue LEP methylation and indicate a better flexibility of progenitor cells and persistence of the methylation pattern in SAT.

In turn, Lesseur et al. found maternal pre-pregnancy obesity associated with lower LEP methylation in both maternal and neonatal cord blood. Consistently, methylation was positively associated between maternal and newborn blood samples. Contrarily, placental methylation expressed a tendency for being higher in newborns of obese women [112]. Later on, American scientists confirmed the link between decreased LEP methylation and increased pre-pregnancy BMI in neonatal cord blood. Moreover, cord blood leptin was positively correlated with newborn birth weight, fat mass, and body fat percentage [117]. Recently, a study distinguishing the fetal and maternal sides of the placenta revealed that the fetal side was more prone to environmentally-induced epigenetic changes than the maternal side. Increased mean LEP methylation in fetal placenta was associated with maternal obesity, which confirms the previous tendency in the placenta. Among the 17 CpG sites analyzed in this study, seven were hypomethylated and four were hypermethylated on both sides of the placenta under conditions of maternal obesity. The authors suggest that various methylation of specific CpGs reflects different regulatory domains of the LEP promoter, which is definitely worth further investigation [118]. Additionally, elevated mean placental LEP methylation seemingly acts as an adaptive mechanism to maternal obesity. In contrary to probable subsequent lower leptin production in the placenta, LEP mRNA expression only tended to be lower and leptin protein level was unchanged [118].

Given the association of cord blood LEP methylation and maternal obesity in previous reports, future studies should answer whether and how placental leptin dysregulation influences the neonatal cord blood epigenome. Maternal obesity-induced changes in cord blood methylation correspond with methylation changes related to LBW, which seems to be impermanent. Thus, in utero memorization mechanisms contribute to increased risk of metabolic diseases in later life, and this occurs at least in early life as for leptin’s epigenetic regulation.

Glucose metabolism disorders during pregnancy are helpful in studying the possible culprits of deleterious fetal programming. A substantial cohort study in 2014 found higher LEP methylation in the placentas associated with pre-pregnancy obesity and GDM, whereas Bouchard et al. indicated that exposure to gestational impaired glucose metabolism affected LEP methylation in the placenta in two distinct manners [119,120]. In the impaired glucose tolerance (IGT) group, the glucose level was positively correlated with the methylation on the maternal side whereas the correlation was negative on the fetal side of the placenta. Accordingly, in the placenta, leptin mRNA levels were negatively correlated with LEP promoter methylation, regardless of its side. Moreover, in both neonatal cord and maternal blood samples, leptin levels were negatively correlated with glucose levels, and in the cord blood of the IGT group, the leptin level was decreased [120].

Antagonistic changes in the methylation pattern between the fetal and maternal side of the placenta suggest the different roles of leptin in regulating energetic metabolism on each side, which needs a particular approach in further research. Nevertheless, leptin levels in both maternal and cord blood indicated a similar correlation to the glucose levels. Leptin levels in third trimester maternal blood samples and cord blood were both significantly decreased. In 2015, Mendelian randomization-based analysis on nearly 500 mother–infant dyads (excluding women treated with insulin or treated for diabetes) showed that cord blood methylation near the LEP gene was associated with fasting plasma glucose (FPG) levels in pregnant woman. Researchers selected CpG of the strongest correlation (negative) with cord blood leptin levels and this was the only CpG whose methylation level was also negatively correlated with maternal FPG [121]. In contrast, a longitudinal study that examined methylation of the LEP promoter in SAT indicated the complexity of prenatally programmed epigenetic regulations of glucose metabolism. Adult offspring of GDM women had increased LEP methylation and lower mRNA expression. Nevertheless, these results require further confirmation seeing that, after adjustment for confounders (e.g., pre-pregnancy BMI) and mediators (such as waist circumference, total body fat percentage, HDL cholesterol), both LEP methylation and expression were unchanged. Furthermore, the lack of association between DNA methylation and plasma leptin or metabolic disease markers only supports the previous notion about initial methylation changes in SAT [122]. Still, GDM-induced changes of the LEP methylation in SAT may contribute to metabolic disorders to some extent. Thus, when distinguishing particular tissue subtypes, DNA methylation changes emerge as different, and seemingly few precise depots and changes may be the main suspects responsible for the fetal programming of metabolic disorders. Next, DNA methylation in both placenta and neonatal cord blood have been negatively correlated with maternal glucose levels. Hence, the identification of intermediate factors or confounders that possibly interfere with the regulation of glucose metabolism between mother and fetus could disentangle this putative interplay.

Finally, research concerning fetal programming in the offspring of pre-pregnancy HFD-fed mice dams also showed changes in DNA methylation and histone modifications near the LEP gene. The study interrogated changes in rat offspring at the age of 12 and 21 postnatal days (PND) and nine months. Methylation of the LEP promoter in SAT was unchanged, however, in the perirenal fat pad (reflecting VAT) of the HFD-fed dams’ offspring, at every stage of study, the methylation of 2 CpGs was lower. Leptin mRNA expression was elevated, whereas inguinal adipose tissue (reflecting SAT) showed increased mRNA only in 12 PND, which was similar to the adipogenesis of SAT. The authors suggest that epigenetic changes of a greater extent affect the LEP gene enhancers (e.g., PPARγ) [123]. On the other hand, undernutrition in mice during pregnancy and lactation also resulted in hypomethylation of the offspring’s LEP promoter, related to a greater postprandial expression of leptin. Despite this, decreased LEP mRNA and leptin levels in the offspring subjected to perinatal undernutrition reflected their lean body composition and lower body weight [124]. Moreover, 60 individuals, whose conception took place during the Dutch famine, exhibited increased blood LEP methylation when compared to unexposed siblings [125]. Interestingly, the association of exposure to famine and LEP methylation was restricted to male subjects [125].

To summarize, rodent model-based studies indicate that both overnutrition and undernutrition may contribute to further metabolic disorders while taking into account that the direction of the changes is similar. Results also imply that visceral adipose tissue could be more prone to deleterious fetal programming than subcutaneous adipose tissue. Elevated leptin expression, even in early postnatal life, may affect epigenetic changes and promote excessive fat accumulation in future life.

On the other hand, several reports have described the role of alternative factors in fetal programming concerning leptin. A cohort study revealed a positive correlation between corticotropin-releasing hormone (CRH) levels and LEP methylation, which persisted in mid-childhood. Since CRH is responsible for the secretion of cortisol, and exposure of which is considered as a factor contributing to the development of metabolic diseases and obesity and influencing fetal growth, there appears to be a question whether DNA methylation is a mediator of fetal programming [126]. Additionally, periconceptional supplemental intake of folic acid altered methylation of LEP promoter methylation in 6-month old infants (negative association) [127]. Recently, the study of rats exposed to dexamethasone, which induced liver steatosis, showed increased methylation of the LEP promoter and DNMT activity along with lower leptin expression in the liver of the treated group. Apart from liver inflammation, researchers implied that changed LEP methylation was a plausible contributory mechanism in chronic liver steatosis [128]. Hence, modifications of the LEP methylation may also appear as an adverse effect of preterm delivery therapy and, thereby, contribute to later life metabolic disorders.

### 7.2. Postranscriptional Histone Modifications

In 2012, Japanese scientists intended to interrogate if HFD contributed to hypertension, IR, or hyperlipidemia and adipokine expression epigenetic changes in the later life of murine offspring exposed to HFD during pregnancy (OH). They reported that methylation of the histone H4 at lysine 20 in the LEP promoter was higher in the WAT of OH, whereas acetyl H3K9 and dimethyl H3K9 was not found to have significantly changed. Concomitantly, elevated leptin and triglycerides (TGs) were associated with elevated LEP mRNA in WAT up to 24 weeks of age. As a result, the HFD-exposed group had worse glucose tolerance along with increased weight and BP when compared to the control group [129]. In further studies, the authors examined results of HFD exposure on the metabolism of female offspring, and whether a normal diet could attenuate putative epigenetic changes. Multigenerational HFD exposure resulted in an accumulation of changes in female offspring, which could be abolished after normal diet treatment in utero for three generations (histone methylation and mRNA expression). Multigenerational effect of HFD in the first two generations induced an increase of monomethyl H4K20 levels at the LEP promoter, the expression of the LEP gene in WAT at 12 and 24 weeks of age, together with an elevation of leptin serum levels [130]. Later on, researchers found that the monomethyl H4K20 level in LEP promoter region was upregulated in offspring with HFD exposure of both parents, yet, still without differences in acetyl and dimethyl H3K9 levels [131]. Results suggest that a histone modification hallmark, made as a consequence of in utero HFD exposure, is acquired over the years and from both parents. Apart from common modifications of H3K9, in this cycle of reports, H4K20 seems to play a pivotal role in regulating the expression of LEP in fetal programming. Nonetheless, recent reports showed changes in histone modifications in the offspring of HFD-fed dams. The At 21 PND lowered trimethylation of H3K9 in leptin promoter persisted in VAT rather than in SAT, as a mark of an opened state for expression that remained after adipogenesis [119]. Although histone acetylation state was mainly unaffected in fetal programming, in 2018, a scientific report found that HDAC activity was elevated by dexamethasone [128]. Finally, fetal programming reflected in histone modifications has focused on changes of histone methylation, however, possible acetylation changes induced by HDAC activity and their role should be elucidated.

### 7.3. miRNAs

Again, miRNAs related to leptin in fetal programming still remain a poorly studied area of research. Recently, Sinha et al. studied a gestational diabetes murine model to investigate the epigenetic regulation of cocaine- and amphetamine-regulated transcript (CART), a gene which was previously found by this research group as a mediator of hyperleptinemia-induced infertility [132,133]. The whole set of combined epigenetic changes include (i) miRNAs, (ii) histone acetylation and methylation, and (iii) altered promoter methylation, switched on the CART promoter into hypersensitive to leptin during in utero fetal programming. Involvement by deregulated glucose metabolism, miR-101, was strictly related to H3K27 acetylation and trimethylation in the CART promoter (Figure 1). Therefore, the authors suggested a plausible model of complex epigenetic regulation of CART expression including concomitant hydroxymethylation of CpG sites [132]. Interestingly, in ovo administration of leptin to hatched broiler chickens resulted in decreased weight with higher liver weight. Moreover, leptin levels in liver and blood were increased. Hepatic TGs and total cholesterol were lower, while their circulating levels increased. Hepatic levels of miRNAs were increased along with the mRNA and protein expression of hepatic lipid metabolism enzymes [134].

To sum up, the first minor research shows miRNA engagement in the fetal programming of leptin. This scientific approach might thoroughly elucidate the processes underlying the programming of metabolic disorders, seeing that miRNAs contribute to the whole machinery regulating gene expression both directly and indirectly, involving other epigenetic mechanisms.

## 8. Epigenetics of Leptin in Early Postnatal Programming

**Figure 3:**
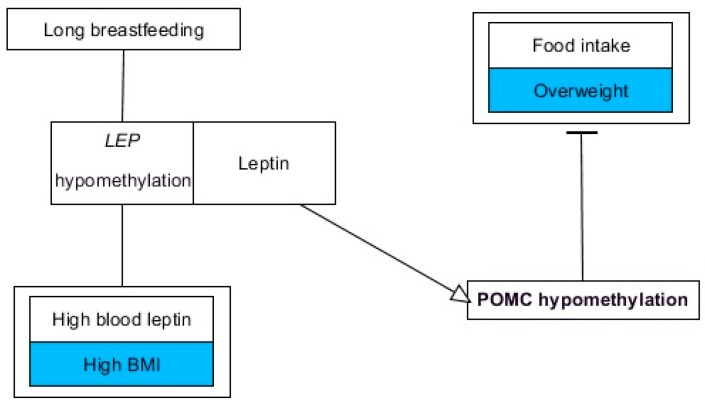
Proposed model of leptin’s participation in perinatal programming. Negative outcomes are highlighted in blue. Associations based on leptin administration are indicated by arrows (stimulation) and T-bars (inhibition). Actions related to boxes refer to all items inside the box. Straight lines indicate correlations. The figure was made using the PathVisio 3.3.0 free open-source software [53].

Aside from fetal programming, early postnatal events are known to contribute to the increased risk of metabolic alteration in later life. One of the mechanisms providing protection against obesity is breastfeeding. This may be due to the fact that breast milk, in contrast to infant formula, contains leptin. Studies have suggested that the leptin level in breast milk has a negative correlation with the body weight of infants until they reached two years old [135]. Rodent studies showed that oral administration of leptin during the entire lactation period was beneficial for neonate rats by protecting them from obesity and overweight [135]. Altogether, breast milk leptin is now considered as a key factor for neonatal development and long-lasting metabolic health programming [136]. Considering the epigenetics of leptin, whole blood LEP methylation was indicated to be negatively correlated with the duration of breastfeeding in very young children (17 months of age), while high leptin concentration and BMI were associated with reduced LEP methylation [137]. Protective effects of leptin were also reported in the study by Palou et al., who used male rats to show that physiological leptin supplementation during their suckling period is capable of affecting methylation status of POMC gene promoter dependent on the diet received (control/HFD) [138]. Impressively, leptin induced programming of an appetite-related gene, thus protecting against adulthood overweight and improving food intake control [138]. Some of the findings regarding the epigenetics of leptin in the context of perinatal programming are depicted in Figure 3.

## 9. Conclusions and Future Perspectives

The results are consistent that the methylation of the LEP promoter is associated with important biochemical and anthropometric parameters, and is engaged in various metabolic disorders. Leptin alone also appears to have an influence on DNA methylation by affecting DNMTs. Deleterious fetal programming might be underlain by a connection between LEP methylation and newborn nutritional status, thus, there a possible relation between the placental and neonatal cord blood leptin dysregulation is of special interest. Investigation of the methylation of particular CpG sites in the LEP promoter showed various outcomes, which could also depend on the stage of obesity development. Therefore, the roles of the methylation status of specific CpGs in metabolic disorder pathogenesis and leptin regulation should be established with regard to the transcription factor binding sites and tissue depots. Clusters of distinguished CpG sites may reflect extensive information, starting from physiological gene expression regulation, long-term history of body nutrition status, hallmarks of pathological processes or their early development, and finally, fetal/early postnatal programming of metabolism. Although a lot of research still needs to be done, it has to be mentioned that DNA methylation is the most well-studied epigenetic mechanism directly regulating leptin, thus far.

Posttranslational histone modifications near the LEP promoter in AT are reported to be responsive to obesity, also playing a role in the regulation of adipogenesis. Nevertheless, leptin’s epigenetic regulation by means of such mechanism remains obscure. Moreover, the dysregulation of leptin was found to involve HDACs and SIRT1 toward the development of obesity, and both HDACs and SIRT1 were indicated as mediators of leptin impact in carcinogenesis. Reports studying posttranslational histone modifications near the LEP promoter are limited and the mechanisms are complex, however, the investigation of suggested early adipogenesis epigenetic events could be a promising direction of research. In particular, in fetal programming, inconsistent histone acetylation diversity requires clarification.

miRNAs have an emerging potential to elucidate the underlying factors of various diseases. Moreover, miRNAs are being favorably investigated in the search for circulating biomarkers of metabolic disorders. These small non-coding RNAs participate in leptin-mediated actions in metabolic processes such as the regulation of POMC, or in the control of adipogenesis by its core regulatory genes, PPARγ and C/EBPα.

Additionally, the participation of miRNAs in the interplay of epigenetic mechanisms regulating leptin signaling, prompt the interrogation of epigenetics in a wider view of interdependent mechanisms. The modulation of leptin signaling and hypothalamic leptin sensitivity suggest that miRNAs may also take part in the dysregulation of these processes. They exert direct and indirect actions on leptin in metabolic imbalance and might play a role in fetal programming, however, the latter still requires more exploration. Finally, the contribution of hyperleptinemia observed in carcinogenesis has resulted in several reports that revealed the involvement of miRNAs, different from those studied in the face of metabolic disorders, in the regulation of leptin signaling. Leptin acts on various transcription factors, thereby affecting epigenetic mechanisms such as miRNAs, posttranslational histone modifications, and modulates DNA methylation through enzymes such as DNMTs. As these mechanisms also influence leptin expression, leptin can be perceived as both their target and mediator. Further research in this area could help to improve therapies of metabolic disorders or even concomitant neoplasms.
